# Forgotten Double-J Ureteral Stent

**DOI:** 10.1590/S1677-5538.IBJU.2019.06.02

**Published:** 2019-12-17

**Affiliations:** 1 Departamento de Litíase e Endourologia da Disciplina de Urologia da Faculdade de Medicina do ABC, Santo André, SP, Brasil

Since the article of Zimsking, 1967, that described the use of a silicon ureteral splint to unblock the renal-ureteral unit, the double J catheter is being routinely used in several urological procedures, particularly in those with obstruction due to urinary stones, urogynecological tumors, urinary stenosis and retroperitoneal fibrosis (1). It can also be used following ureteral lesions or to reduce the risk of inadvertent trauma during complex abdominal and pelvic surgeries (2). Many patients are treated only with the double J catheter and in others the drainage of the urinary system may postpone definitive treatment for a better moment, particularly in the presence of infection.

There are some negative side effects of its use such as discomfort and alteration of micturition, hematuria, perineal and genital pain, and the occurrence of urinary infections or even pyelonephritis (3). New catheters are being developed, with different designs, width, length, material, flexibility, in order to minimize these symptoms. Other clinical studies have evaluated drugs that can ameliorate the clinical setting, such as anticholinergics, alpha-blockers and analgesics.

However, more severe complications are observed with forgotten catheters at the urinary system, that encrust, form stones, fragment, “stenturia” and encrust with obstruction and loss of renal function (4, 5-7). These cases are complex and require multiple endo-urological procedures in order to remove the catheter and the associated stones, including shock wave lithotripsy, percutaneous surgery, ureteral and renal lithotripsy, or even nephrectomies (6-8). Lam et al. analyzed 26 forgotten catheters and showed that it was necessary the use of 2.7 procedures to resolve the situation (9). Forgotten double J stents and their complications are not rare in literature (4-8, 10, 11) particularly in public health services and lower income patients, due to the difficulty to follow up this population that are unaware of the severity of the condition. Likewise, Divakaruni et al observed 16% of forgotten double J catheters in their retrospective cohort, and identified a higher risk group including males and patients without medical insurance (2.5 and 6 times more prone to this complication, respectively) (11). The professional that inserts the catheter must be aware of the follow up of those patients and must certify that the catheter is removed on time, according to good medical practices. In literature, it is observed that the median time for complications of indwelling catheter at the urological system is 3-24 months (3-5, 12). El-Faqih showed that the rate of complications was 9.6% when the catheter was removed in up to 6 weeks following implantation. When maintained for 6 to 12 weeks, this rate rises to 47.5% and for more time to 76.3% (13). Similar figures were observed by Kawahara; incrustation was observed in 26.8% before 6 weeks and reached 75.9% when maintained for more than 12 weeks (5). However, patients characteristics such as hipercalciuria, pregnancy or severe predisposition to stones may lower this time.

Aside from clinical complications as exposed, we must be aware of legal implications. Professionals may suffer lawsuits; patients may feel that they were harmed for not being correctly informed about the need of catheter removal or even being unaware that they were carrying one. Duty et al. revised malpractice litigations from 2005 to 2010 and found 585 urological complaints. Among them, 25 (4.3%) were related to endourological procedures and 4 due to forgotten double J catheter. Osmal et al revised the lawsuits of the British Health System from 1995 to 2009, and found 13% complaints related to forgotten ureteral stents, more frequently at post-operatory (14). Prevention is the better alternative for this situation, in spite of the many efficient endourological treatments. The use of a fixed wire at the distal end of the catheter exteriorized at the urethra eases removal and minimizes forgetfulness. Follow up procedures of patients with double J stents were proposed in order to control these patients by cell phones, computer software and warning systems of patients and doctors (15-18). Sancaktukar et al. performed a randomized study and showed that the group that was followed by SMS for catheter withdrawal was statistically efficient (16). Regardless of which method, it is fundamental that patients with a double J catheter must be monitored and followed-up, particularly in services with high number of procedures and professionals, such as public health services and medical residency programs. The use of warning systems and other similar technologies are more efficient than written forms or cards delivered to patients, since up to 25% of them were not registered (19,20).

Forgotten ureteral stents may cause only mild symptoms or even loss of renal function. After catheter implantation, patients must be well monitored to avoid discontinuity of treatment and maintenance of the catheter for more time than needed. The professional is responsible for follow-up and removal of catheter at the correct time, avoiding related complications.

## References

[1] 1. Zimskind PD, Fetter TR, Wilkerson JL. Clinical use of long-term indwelling silicone rubber ureteral splints inserted cystoscopically. J Urol. 1967;97:840-4. 10.1016/S0022-5347(17)63130-6 6025928

[2] 2. Kuno K, Menzin A, Kauder HH, Sison C, Gal D. Prophylactic ureteral catheterization in gynecologic surgery. Urology. 1998;52:1004-8. 10.1016/s0090-4295(98)00382-3 9836545

[3] 3. Monga M, Klein E, Castañeda-Zúñiga WR, Thomas R. The forgotten indwelling ureteral stent: a urological dilemma. J Urol. 1995;153:1817-9. 7752325

[4] 4. Zahran MH, Harraz AM, Taha DE, El-Nahas AR, Elshal A, Shokeir AA. Studying the Morbidity and Renal Function Outcome of Missed Internal Ureteral Stents: A Matched Pair Analysis. J Endourol. 2015;29:1070-5. 10.1089/end.2015.0047 25793431

[5] 5. Kawahara T, Ito H, Terao H, Yoshida M, Matsuzaki J. Ureteral stent encrustation, incrustation, and coloring: morbidity related to indwelling times. J Endourol. 2012;26:178-82. 10.1089/end.2011.0385 22007916

[6] 6. Adanur S, Ozkaya F. Challenges in treatment and diagnosis of forgotten/encrusted double-J ureteral stents: the largest singlecenter experience. Ren Fail. 2016;38:920-6. 10.3109/0886022X.2016.1172928 27089423

[7] 7. Rana AM, Sabooh A. Management strategies and results for severely encrusted retained ureteral stents. J Endourol. 2007;21:628-32. 10.1089/end.2006.0250 17638560

[8] 8. Mohan-Pillai K, Keeley FX Jr, Moussa SA, Smith G, Tolley DA. Endourological management of severely encrusted ureteral stents. J Endourol. 1999;13:377-9. 10.1089/end.1999.13.377 10446801

[9] 9. Lam JS, Gupta M. Tips and tricks for the management of retained ureteral stents. J Endourol. 2002;16:733-41. 10.1089/08927790260472881 12542876

[10] 10. Goel HK, Kundu AK, Maji TK, Pal DK. Retained fragmented double J ureteric stent: A report of four cases with review of the literature. Saudi J Kidney Dis Transpl. 2015;26:747-50. 10.4103/1319-2442.160199 26178549

[11] 11. Divakaruni N, Palmer CJ, Tek P, Bjurlin MA, Gage MK, Robinson J, et al. Forgotten ureteral stents: who's at risk? J Endourol. 2013;27:1051-4. 10.1089/end.2012.0754 23590526

[12] 12. Singh V, Srinivastava A, Kapoor R, Kumar A. Can the complicated forgotten indwelling ureteric stents be lethal? Int Urol Nephrol. 2005;37:541-6. 10.1007/s11255-004-4704-6 16307339

[13] 13. el-Faqih SR, Shamsuddin AB, Chakrabarti A, Atassi R, Kardar AH, Osman MK, et al. Polyurethane internal ureteral stents in treatment of stone patients: morbidity related to indwelling times. J Urol. 1991;146:1487-91. 10.1016/s0022-5347(17)38146-6 1942324

[14] 14. Osman NI, Collins GN. Urological litigation in the UK National Health Service (NHS): an analysis of 14 years of successful claims. BJU Int. 2011;108:162-5. 10.1111/j.1464-410X.2011.10130.x 21481130

[15] 15. Ziemba JB, Ludwig WW, Ruiz L, Carvalhal E, Matlaga BR. Preventing the Forgotten Ureteral Stent by Using a Mobile Point-of-Care Application. J Endourol. 2017;31:719-24. 10.1089/end.2017.0118 28443681

[16] 16. Sancaktutar AA, Tepeler A, Söylemez H, Penbegül N, Atar M, Bozkurt Y, et al. A solution for medical and legal problems arising from forgotten ureteral stents: initial results from a reminder short message service (SMS). Urol Res. 2012;40:253-8. 10.1007/s00240-011-0404-8 21792673

[17] 17. Ulker V, Atalay HA, Cakmak O, Yucel C, Celik O, Kozacioglu Z. Smartphone-based stent tracking application for prevention of forgotten ureteral double-J stents: a prospective study. Int Braz J Urol. 2019;45:376-83. 10.1590/S1677-5538.IBJU.2018.0707 PMC6541122 30785702

[18] 18. Molina WR, Pessoa R, Donalisio da Silva R, Kenny MC, Gustafson D, Nogueira L, et al. A new patient safety smartphone application for prevention of “forgotten” ureteral stents: results from a clinical pilot study in 194 patients. Patient Saf Surg. 2017;11:10. 10.1186/s13037-017-0123-3 PMC5381069 28396695

[19] 19. Tang VC, Gillooly J, Lee EW, Charig CR. Ureteric stent card register - a 5-year retrospective analysis. Ann R Coll Surg Engl. 2008;90:156-9. 10.1308/003588408X242123 PMC2443315 18325220

[20] 20. Thomas AZ, Casey RG, Grainger R, McDermott T, Flynn R, Thornhill JA. The forgotten ureteric JJ stent and its prevention: a prospective audit of the value of a ureteric stent logbook. Ir J Med Sci. 2007;176:117-9. 10.1007/s11845-007-0043-9 17516130

